# The long-acting C5 inhibitor, ravulizumab, is efficacious and safe in pediatric patients with atypical hemolytic uremic syndrome previously treated with eculizumab

**DOI:** 10.1007/s00467-020-04774-2

**Published:** 2020-10-13

**Authors:** Kazuki Tanaka, Brigitte Adams, Alvaro Madrid Aris, Naoya Fujita, Masayo Ogawa, Stephan Ortiz, Marc Vallee, Larry A. Greenbaum

**Affiliations:** 1 Department of Nephrology, Aichi Children’s Health and Medical Center, 7-426, Morioka-cho, Obu City, Aichi prefecture 474-8710 Japan; 2 Department of Pediatric Nephrology, Children’s Hospital Queen Fabiola, Université libre de Bruxelles, Brussels, Belgium; 3 Children’s Nephrology and Renal Transplantation Service, Children’s Maternity Hospital Sant Joan de Déu, University of Barcelona, Barcelona, Spain; 4 Alexion Pharmaceuticals Inc, Boston, MA USA; 5 Division of Pediatric Nephrology, Emory University School of Medicine and Children’s Healthcare of Atlanta, Atlanta, GA USA

**Keywords:** Atypical hemolytic uremic syndrome, Complement, Eculizumab, Hemolytic uremic syndrome, Ravulizumab, Thrombotic microangiopathy, Children

## Abstract

**Background:**

Atypical hemolytic uremic syndrome (aHUS) is a rare, complement-mediated disease associated with poor outcomes if untreated. Ravulizumab, a long-acting C5 inhibitor developed through minimal, targeted modifications to eculizumab was recently approved for the treatment of aHUS. Here, we report outcomes from a pediatric patient cohort from the ravulizumab clinical trial (NCT03131219) who were switched from chronic eculizumab to ravulizumab treatment.

**Methods:**

Ten patients received a loading dose of ravulizumab on Day 1, followed by maintenance doses administered initially on Day 15, and then, every 4–8 weeks thereafter, depending on body weight. All patients completed the initial evaluation period of 26 weeks and entered the extension period.

**Results:**

No patients required dialysis at any point throughout the study. The median estimated glomerular filtration rate values remained stable during the trial: 99.8 mL/min/1.73m^2^ at baseline, 93.5 mL/min/1.73m^2^ at 26 weeks, and 104 mL/min/1.73m^2^ at 52 weeks. At last available follow-up, all patients were in the same chronic kidney disease stage as recorded at baseline. Hematologic variables (platelets, lactate dehydrogenase, and hemoglobin) also remained stable throughout the initial evaluation period and up to the last available follow-up. All patients experienced adverse events; the most common were upper respiratory tract infection (40%) and oropharyngeal pain (30%). There were no meningococcal infections reported, no deaths occurred, and no patients discontinued during the study.

**Conclusions:**

Overall, treatment with ravulizumab in pediatric patients with aHUS who were previously treated with eculizumab resulted in stable kidney and hematologic parameters, with no unexpected safety concerns when administered every 4–8 weeks.

**Trial registration:**

Trial identifiers:

Trial ID: ALXN1210-aHUS-312

Clinical trials.gov: NCT03131219

EudraCT number: 2016-002499-29

**Figure:**
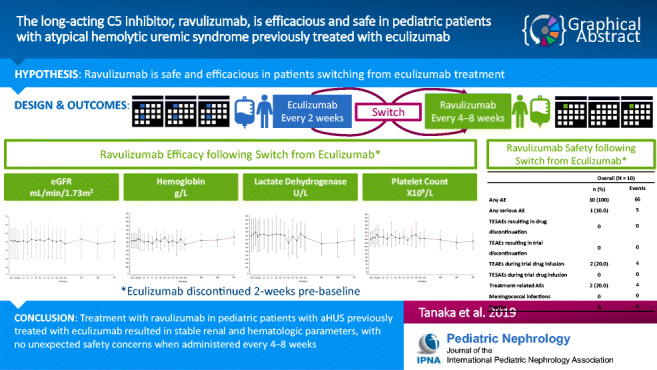
Graphical abstract

**Electronic supplementary material:**

The online version of this article (10.1007/s00467-020-04774-2) contains supplementary material, which is available to authorized users.

## Introduction

Atypical hemolytic uremic syndrome (aHUS) is a rare, progressive disease caused by uncontrolled complement activation [1–3]. The endothelial damage caused by thrombotic microangiopathy (TMA) can result in life-threatening manifestations of the disease, including kidney failure and extrarenal tissue damage [1]. Prior to the availability of targeted treatment with complement C5 inhibitors, the prognosis of aHUS both in pediatric and adult patients was poor—around 29% of children required dialysis or died within 1 year [4] and 48% reached chronic kidney disease (CKD) stage 5 or death at 3 years despite plasma therapy [5].

The complement protein C5 inhibitor eculizumab (Soliris®, Alexion Pharmaceuticals, Inc., Boston, MA, United States) has been shown to be efficacious and safe in treating aHUS in adults and children over the last decade, both in clinical trials and real-world settings [6–13]. However, despite its proven effectiveness, intravenous (IV) eculizumab infusions are required once every 2 weeks, which may be burdensome for patients and caregivers.

Ravulizumab is a humanized monoclonal antibody (mAb) engineered from eculizumab to target the same complement C5 epitope while decreasing drug clearance and reducing the required frequency of infusions [14]; a schematic detailing the specific amino acid substitutions and the mechanism of half-life prolongation can be found in Supplementary Fig. 1. Ravulizumab incorporates selective amino acid modifications to both increase the dissociation rate of the mAb:C5 complex in the acidic early endosome at pH 6.0 and enhance the efficiency of neonatal Fc receptor-mediated antibody recycling, leading to an extended duration of terminal complement inhibition [14]. Ravulizumab has a mean terminal elimination half-life that is over 4 times greater than that of eculizumab (~ 51.8 days vs. ~ 11 days) and offers a reduced dosing frequency of 4–8 weeks vs. every 2–3 weeks, depending on bodyweight [14].

Ravulizumab has recently been approved for the treatment of aHUS in adults and children [15, 16]. The clinical efficacy and safety of ravulizumab in complement inhibitor-naive adults with aHUS have been demonstrated in a recently published phase III trial report (NCT02949128) [17], and a similar report detailing results in pediatric patients from the treatment-naive cohort of the current trial (NCT03131219) is underway; eculizumab has previously been shown to be efficacious and safe in pediatric patients with aHUS [8, 10]. Here, we assessed the efficacy of ravulizumab through 50 weeks of follow-up and safety through all available follow-up in pediatric patients with aHUS, with stable TMA parameters following a switch from eculizumab to ravulizumab treatment.

## Methods

### Trial oversight and design

ALXN1210-aHUS-312 (NCT03131219) is a phase III, single-arm, multicenter trial assessing the efficacy and safety of ravulizumab administered by IV infusion in pediatric patients (< 18 years of age) with aHUS who are either naive to complement inhibitor treatment (cohort 1, reported separately) or eculizumab-treated (cohort 2, this analysis). The protocol was approved by the Institutional Review Board (IRB) or Independent Ethics Committee (IEC) at each participating center, and the trial was conducted in accordance with the Declaration of Helsinki and the Council for International Organizations of Medical Sciences International Ethical Guidelines. The patient’s legal guardians were required to provide a written informed consent and the patient written informed assent (if applicable, as determined by the central or local IRB or IEC).

The trial consisted of a screening period (up to 28 days), a 26-week initial evaluation period, and an extension period (up to 4.5 years). After completion of the initial evaluation period, patients were eligible to enroll in the extension period, which remains ongoing. Here, we present data up to 59 weeks of follow-up.

**Fig. 1:**
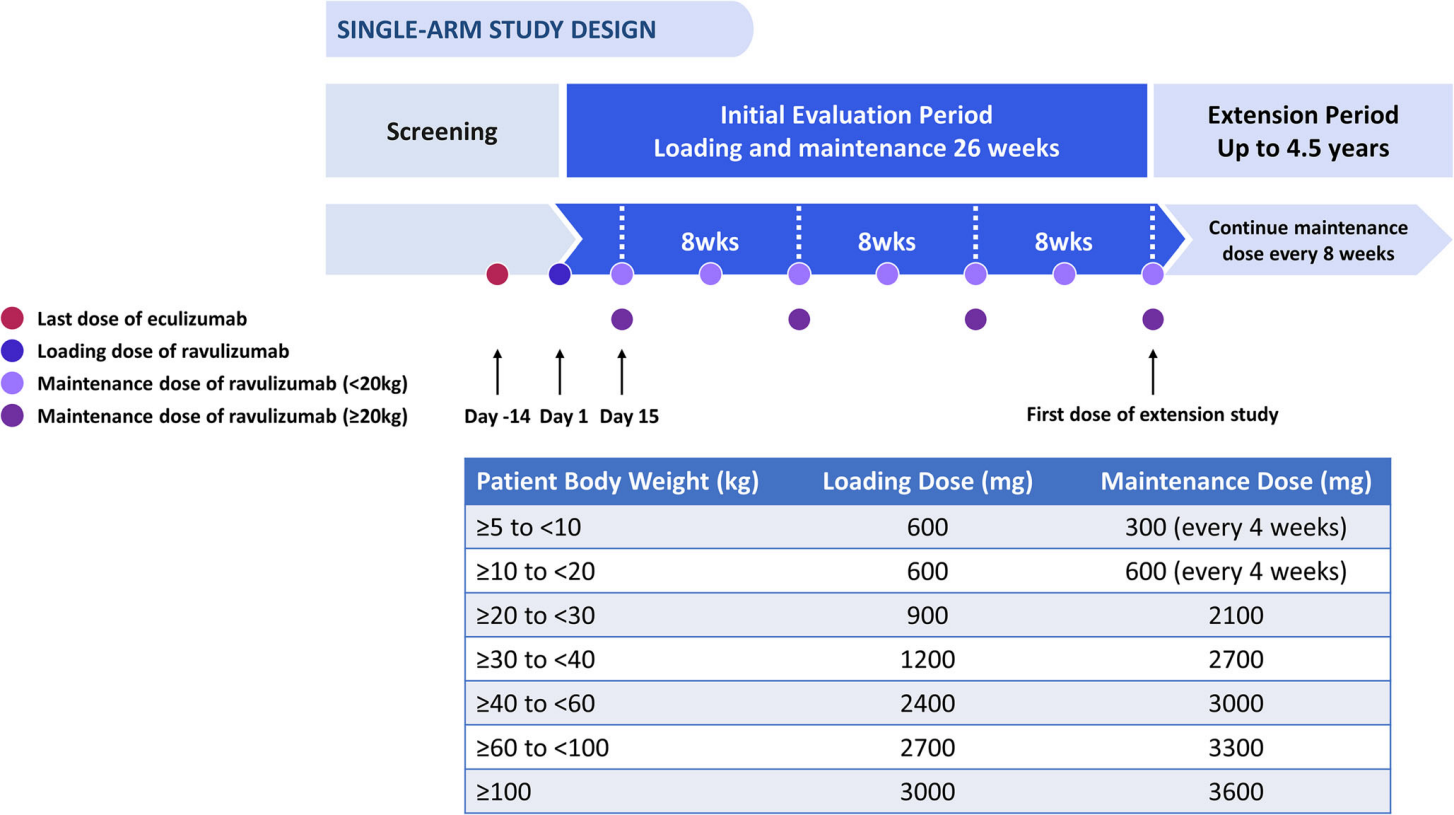
Study design with dosing schedule

Ravulizumab was administered via IV infusion based on bodyweight (Fig. 1) with an infusion time of approximately 2 h. On Day 1 of the initial evaluation period, which was 14 days from the patient’s last dose of eculizumab, IV loading doses of ravulizumab were administered. Starting on Day 15, maintenance doses were administered every 8 weeks in patients weighing ≥ 20 kg and every 4 weeks in patients < 20 kg (Fig. 1). Baseline was defined as the period between screening and the point of first drug infusion (Day 1, inclusive).

### Patients

The full inclusion and exclusion criteria for the phase III pediatric ravulizumab clinical trial can be found in Supplementary Table 1. Patients included in this specific cohort analysis were male or female, < 18 years of age and ≥ 5 kg at the time of consent; patients must also have been treated with eculizumab according to the labelled dosing recommendation for aHUS for at least 90 days prior to screening. Clinical evidence of response to eculizumab was indicated by stable TMA parameters at screening, including lactate dehydrogenase (LDH) < 1.5 × upper limit of normal, platelet count ≥ 150,000/μL, and estimated glomerular filtration rate (eGFR) > 30 mL/min/1.73m^2^ using the Schwartz formula [18].

All patients being transitioned to ravulizumab therapy were required to have up-to-date vaccinations against *Neisseria meningitidis* in line with all local guidelines and regulations, irrespective of previous vaccination prior to initiation of eculizumab.

Patients excluded from this study included those with a familial or acquired deficiency in A disintegrin and metalloproteinase with a thrombospondin type 1 motif, member 13 (ADAMTS13; activity < 5%); Shiga toxin–related hemolytic uremic syndrome; positive direct Coombs test; drug exposure–related HUS; bone marrow transplant/hematopoietic stem cell transplant within last 6 months prior to start of screening; known genetic defects of cobalamin C metabolism; scleroderma; systemic lupus erythematosus; or antiphospholipid antibody positivity syndrome. Patients on chronic dialysis (defined as dialysis on a regular basis as kidney replacement therapy for kidney failure) were also excluded.

### Genetic testing and factor H autoantibody assessment

Exploratory analysis of pathogenic complement genetic variants was performed in consenting patients using whole-exome sequencing on the NovaSeq 6000 platform with a 2 × 150-bp paired-end molecule (Illumina, Inc., San Diego, CA). Pathogenesis was determined as shown in the supplementary whole-exome sequencing and assessment of variant pathogenicity process (Supplementary Table 2). Exploratory assessments of the presence of autoantibodies to complement proteins, such as complement factor H (CFH), were also performed. Assessment of anti-CFH autoantibodies was based upon the assay described by Dragon-Durey et al. [19].

### Efficacy endpoints

The endpoints for this cohort included dialysis requirement status; observed value and change from baseline in eGFR; CKD stage [20] (as evaluated by eGFR at select target days and classified as improved, stable [no change], or worsened compared with baseline); observed value and change from baseline in hematologic parameters (platelets, LDH, hemoglobin); change from baseline in quality of life, as measured by pediatric Functional Assessment of Chronic Illness Therapy-Fatigue (FACIT-Fatigue) scale; and TMA parameters in patients who discontinued treatment in the extension period but remained in the trial. Efficacy data is presented up to 50 weeks of follow-up.

### Pharmacokinetic/pharmacodynamic endpoints

Pharmacokinetic (PK)/pharmacodynamic (PD) endpoints included changes in serum ravulizumab concentrations over time and changes in serum free C5 concentrations (a measure of terminal complement inhibition) over time, respectively. PK/PD analyses were performed in all patients with evaluable data who had received ≥ 1 dose of ravulizumab.

### Safety endpoints

The long-term safety and tolerability of ravulizumab was evaluated by physical examinations, vital signs, physical growth (height), electrocardiograms, laboratory assessments, and incidence, severity, and seriousness of adverse events (AEs). The severity of AEs was graded using the Common Terminology Criteria for Adverse Events (CTCAE) version 4.03 and coded using the Medical Dictionary for Regulatory Activities (MedDRA®) version 21.0. The proportion of patients who developed antidrug antibodies was also assessed. Safety data is presented up to 59 weeks of follow-up.

### Statistical analyses

Efficacy analyses were performed on the full analysis set, including all patients who received ≥ 1 dose of ravulizumab with at least 1 post-baseline efficacy assessment. eGFR value and change from baseline were summarized using descriptive statistics. The proportion of patients who no longer required dialysis was evaluated with a 2-sided 95% confidence interval. CKD stage was summarized over time presenting the number and proportion of patients who improved, worsened, or stayed the same compared with CKD stage at baseline [20]. Platelets, LDH, hemoglobin, and FACIT-Fatigue were summarized using descriptive statistics for observed values and change from baseline. Quality of life was assessed via the FACIT-Fatigue scale and results were summarized using descriptive statistics for both continuous variables at baseline and all post-baseline visits, alongside change from baseline.

Safety analyses were performed for the safety set, defined as all patients who received ≥ 1 dose of ravulizumab. All AEs were summarized by organ system class and preferred term. Abnormal immunogenicity findings were presented descriptively over time.

## Results

### Patient characteristics

A total of 10 eculizumab-treated pediatric patients with a diagnosis of aHUS who met the trial criteria were enrolled and received ≥ 1 dose of ravulizumab. Patients in this cohort had a median age of 12.5 (range, 1.2–15.5) years and were predominantly male (9 patients (90%); Table 1). Five (50%) patients were white, with the remaining patients either Asian (40%) or Black/African American (10%). No patients were on dialysis at baseline and none had received plasma exchange/infusion prior to receiving ravulizumab. No patients had received prior bone marrow transplantation and one patient had a prior kidney transplantation directly related to their diagnosis of aHUS. Patients had received eculizumab for between 98 and 1701 days. At the time of first infusion, patients had a median weight of 47.8 (range, 8.8–69.0) kg.

**Table 1:** Baseline demographics, disease characteristics, and laboratory values

Variable	Overall (*N* = 10)
Median age at time of first infusion (range), years	12.5 (1.2–15.5)
Age at time of first infusion (years) category	
Birth to < 2 years	1 (10.0)
2 to < 6 years	1 (10.0)
6 to < 12 years	1 (10.0)
12 to < 18 years	7 (70.0)
Sex	
Male	9 (90.0)
Female	1 (10.0)
Race	
Asian	4 (40.0)
Black or African American	1 (10.0)
White	5 (50.0)
Median weight at time of first infusion (range), kg	47.8 (8.82–69)
Weight at time of first infusion category	
≥ 5 to < 10 kg	1 (10.0)
≥ 10 to < 20 kg	1 (10.0)
≥ 20 to < 30 kg	1 (10.0)
≥ 30 to < 40 kg	1 (10.0)
≥ 40 to < 60 kg	5 (50.0)
≥ 60 kg	1 (10.0)
Any prior kidney transplant^a^	1 (10.0)
Plasma exchange/infusion prior to trial drug	0
Kidney dialysis within 56 days prior to trial drug	0
Median platelet count (range), × 10^9^/L	281.75 (207–415.5)
Median LDH (range), U/L	206.50 (138.5–356)
Median serum creatinine (range), μmol/L	50.75 (23.5–111.5)
Median eGFR (range), mL/min/1.73 m^2^	99.75 (54–136.5)
Median hemoglobin (range), g/L	132.00 (114.5–148)

Data shown as *n* (%) unless otherwise stated. ^a^Kidney transplant was related to aHUS

*aHUS* atypical hemolytic uremic syndrome, *eGFR* estimated glomerular filtration rate, *LDH* lactate dehydrogenase, *SD* standard deviation

Four patients underwent genetic testing as part of this study, of whom 2 were found to possess pathogenic variants in complement genes; 1 patient was found to possess nonsense variant c.175C > T (p.Arg59Ter) in the membrane cofactor protein (*MCP*, *CD46*) gene and the other possessed missense variant c.3644G > A (p.Arg1215Gln) in the *CFH* gene. Both of these genetic variants have been previously reported in patients with aHUS [21, 22]. One patient was found to have detectable levels of CFH autoantibodies. Additional pathogenic variants in these patients have been identified following local genetic analyses (Supplementary Table 2).

All 10 patients completed the 26-week initial evaluation period and entered the extension period. None discontinued ravulizumab or the trial. At the current data-cut, at a median duration of 50.3 (range, 49.4–58.7) weeks, all 10 patients were still enrolled in the extension period.

### Kidney endpoints

**Fig. 2:**
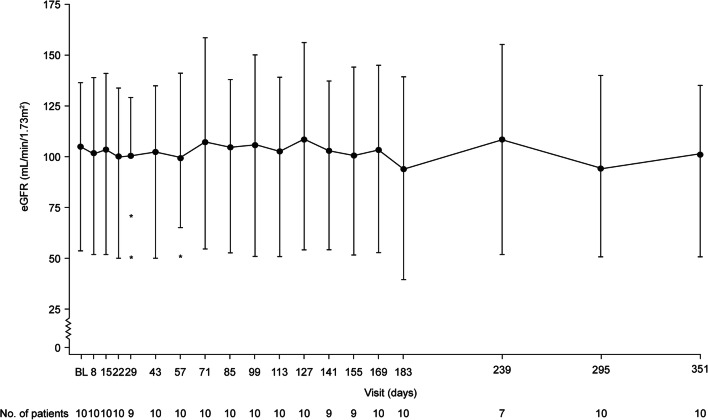
Change in eGFR over time, for the initial evaluation and the extension period (full analysis set). *eGFR* estimated glomerular filtration rate

None of the 10 patients were undergoing dialysis at baseline and no patients required dialysis at any point post-ravulizumab treatment during the trial. Kidney function, measured by eGFR values over time, remained stable throughout the trial. The recorded median values were 99.8 mL/min/1.73m^2^ (range, 54.0–136.5) at baseline, 93.5 mL/min/1.73m^2^ (range, 40.0–139.0) at 26 weeks, and 104 mL/min/1.73m^2^ (range, 51.0–135.0) at 1 year (Fig. 2). At baseline, 8 patients (80.0%) were at CKD stage 1 (≥ 90 mL/min/1.73m^2^), 1 patient (10.0%) at CKD stage 2 (60–89 mL/min/1.73m^2^), and 1 patient (10.0%) was at CKD stage 3a (45–59 mL/min/1.73m^2^). At 1 year, all patients remained within the same CKD stage as observed at baseline (Supplementary Fig. 2).

### Hematologic endpoints

**Fig. 3:**
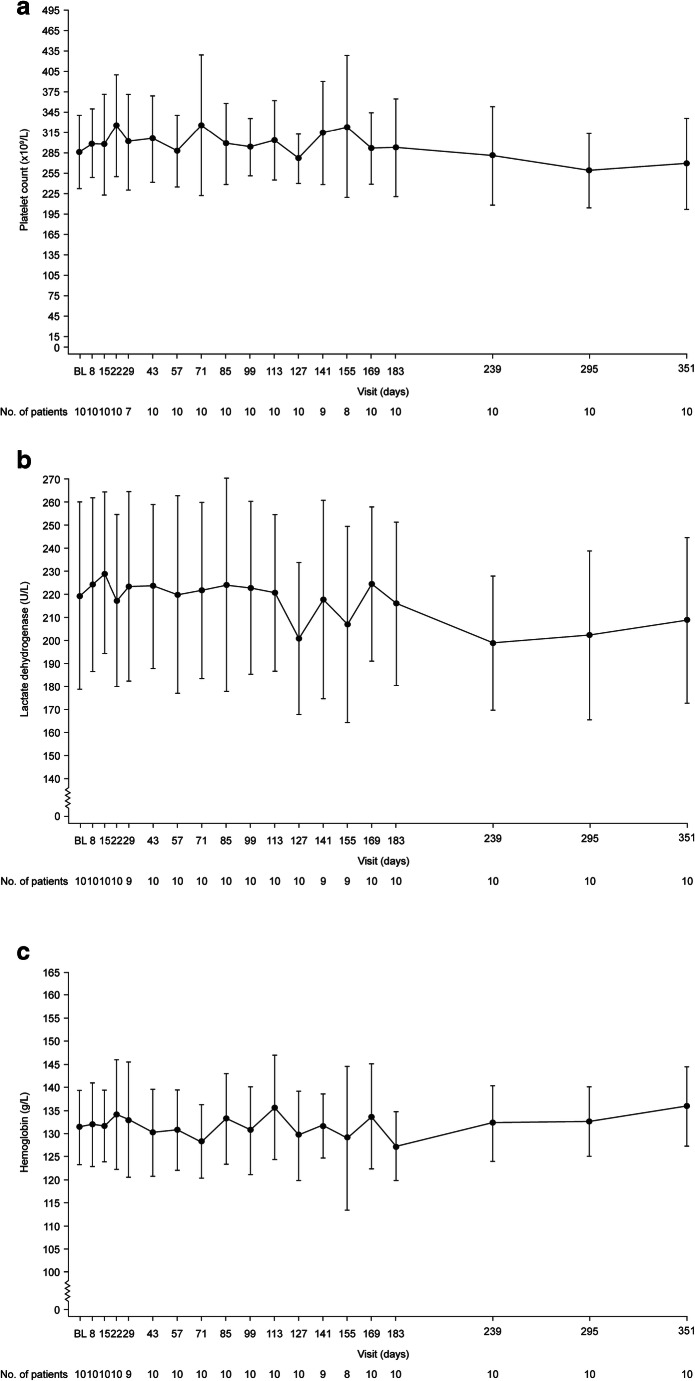
Mean (95% CI) observed values over time during the initial evaluation period and the extension period (full analysis set) for platelet count (**a**), lactate dehydrogenase (LDH) (**b**), and hemoglobin (**c**). *CI* confidence interval, *LDH* lactate dehydrogenase

Hematologic parameters remained stable throughout the 26-week initial evaluation period and up to 1 year of the extension period (Fig. 3). The median (range) change in platelet count from baseline to 26 weeks and from baseline to 1 year was 2.3 (− 74.5, 123.5) 10^9^/L and − 34.8 (− 109.0, 109.0) 10^9^/L, respectively; the median change in LDH was 8.5 (− 50.5, 50.5) and − 17.5 (− 34.5, 29.5), respectively; and the median change in hemoglobin was 3.5 (− 19.5, 8.0) and 5.5 (− 7.5, 13.5), respectively.

### Quality of life endpoints

The FACIT-Fatigue scores remained stable throughout the initial evaluation period and extension period up to 1 year (Supplementary Fig. 3). The median (range) FACIT-Fatigue value at baseline was 50.0 (42.0, 52.0). The median change from baseline was 0.0 (− 5.0, 3.0) at 26 weeks and − 1.0 (− 7.0, 2.0) at 1 year.

### PK/PD analysis

**Fig. 4:**
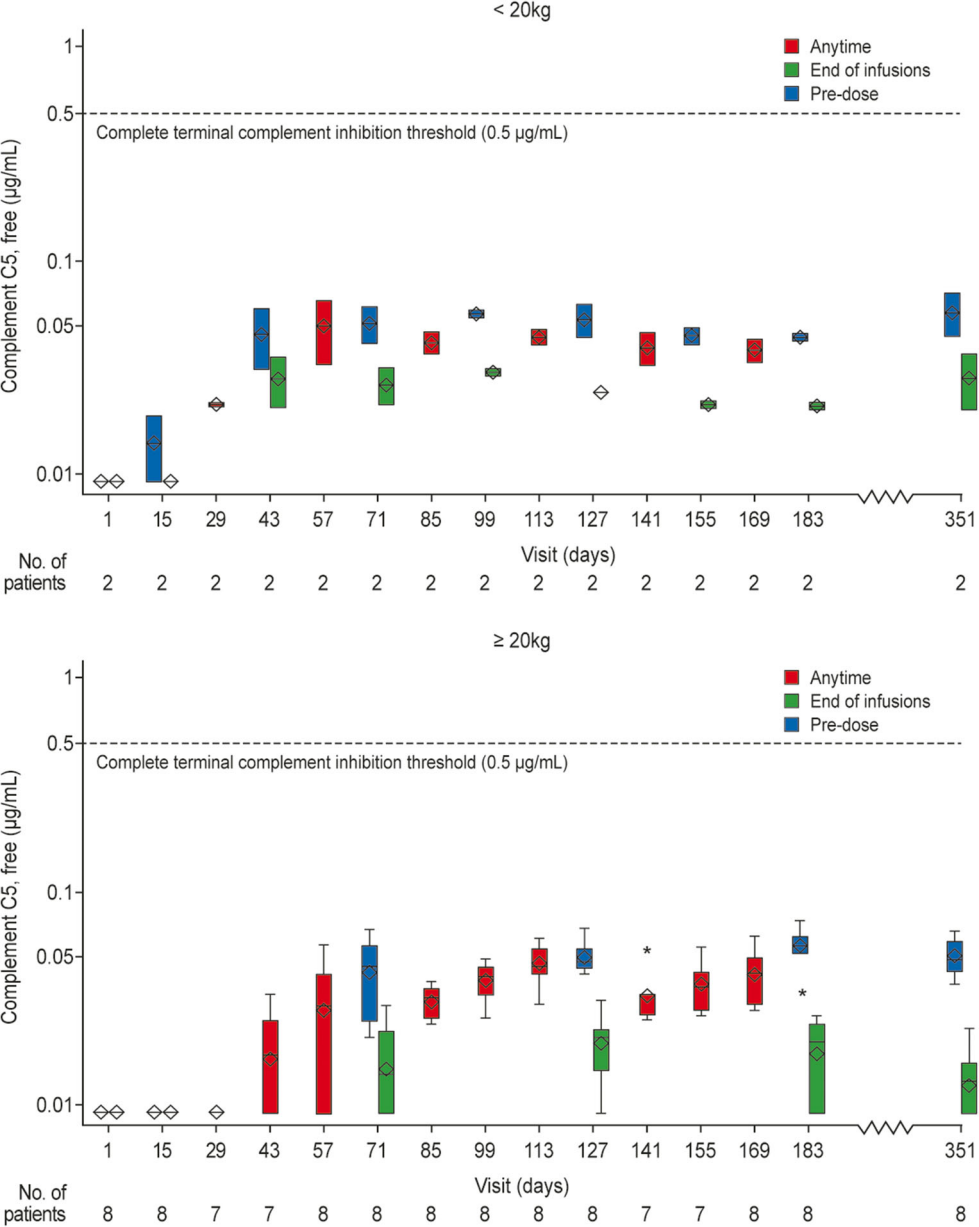
Free C5 concentrations over time (PK/PD analysis set). A dashed line is drawn at 0.5 ug/ml free C5 to denote the threshold for complete terminal complement inhibition. The horizontal line in the middle of each box indicates the median, a diamond indicates the mean, and the top border and the bottom border of the boxes mark the 75th and 25th percentiles, respectively. The whiskers represent the highest and lowest values within 1.5 times the interquartile range from the lower quartile and upper quartile. Outliers are represented by an asterisk beyond the whiskers. Patients weighing < 20 kg (**a**) and patients weighing ≥ 20 kg (**b**). *PD* pharmacodynamic, *PK* pharmacokinetic

The steady-state therapeutic serum ravulizumab concentrations were achieved immediately following treatment initiation, with steady-state maximal and trough exposures observed as expected following dosing (Supplementary Fig. 4). Weight-based dosing of ravulizumab resulted in immediate, complete, and sustained terminal complement inhibition (defined as serum free C5 concentrations of < 0.5 μg/mL; Fig. 4).

### Safety analyses

All 10 (100%) patients in this analysis experienced AEs (Table 2). One (10%) patient experienced an AE of grade 3; all others were of grades 1 and 2. Five serious AEs related to respiratory tract infections were recorded in 1 (10%) patient (3 upper respiratory tract infections, 1 instance of bronchitis, and 1 instance of pneumonia), none of which were considered treatment related; all AEs in this patient were resolving and did not require drug or study discontinuation (Table 2). Overall, the most frequent treatment-emergent AEs (Table 3) were upper respiratory tract infection (40%) and oropharyngeal pain (30%). All other AEs were reported by ≤ 2 patients.

**Table 2:** Summary of adverse events through the current data-cut^a^ (safety set)

	Overall (*N* = 10)	
	*n* (%)	Events
Any AE	10 (100)	66
Any serious AE	1 (10.0)	5
TESAEs resulting in drug discontinuation	0	0
TEAEs resulting in trial discontinuation	0	0
TEAEs during trial drug infusion	2 (20.0)	4
TESAEs during trial drug infusion	0	0
Treatment-related AEs	2 (20.0)	4
Meningococcal infections	0	0
Deaths	0	0

^a^Current data-cut at median follow-up duration of 50.2 weeks

AEs were coded using the MedDRA version 21.0. The severity of AEs was graded using the CTCAE version 4.03

*AE* adverse event, *TEAE* treatment-emergent adverse event, *TESAE* treatment-emergent serious adverse event

**Table 3:** Most frequent treatment-emergent adverse events

	Overall (*N* = 10)	
	*n* (%)	Events
Upper respiratory tract infection	4 (40.0)	19
Nasopharyngitis	2 (20.0)	2
Otitis media	2 (20.0)	2
Pharyngitis	2 (20.0)	2
Viral upper respiratory tract infection	2 (20.0)	2
Oropharyngeal pain	3 (30.0)	3

Events occurring in > 15% of patients listed. Adverse event terms are as reported by the treating investigator. Patients evaluated for safety included all patients that received ≥ 1 dose of the study drug

Treatment-related AEs (dyspepsia and musculoskeletal pain) were reported by 2 (20.0%) patients. The dyspepsia began and resolved on trial Day 1 with no change to trial treatment. The patient with musculoskeletal pain experienced this AE twice: on Day 15 and on Day 71. On Day 15, the AE resolved the same day with dose interruption. On Day 71, the AE resolved the same day with no action taken with trial treatment. There were no clinically significant findings reported in physical examinations, vital signs, growth (height), electrocardiograms, or laboratory investigations. No patients had a positive antidrug antibody titer recorded during the trial. No meningococcal infections or deaths were reported during the trial.

## Discussion

This manuscript presents the analysis of a cohort of patients switching from eculizumab to ravulizumab treatment, taken from the first prospective phase III trial of ravulizumab, a long-acting C5 inhibitor in pediatric patients. Data from this trial were analyzed in 2 cohorts: complement inhibitor-naive pediatric patients (analysis published separately) and pediatric patients switching from eculizumab therapy (current analysis). The current analysis shows that following induction dosing, terminal complement inhibition (serum complement free C5 concentrations <0.5 μg/ml), kidney function, and hematological parameters remained stable when patients transitioned from eculizumab to weight-based maintenance dosing of ravulizumab every 4–8 weeks. Mean eGFR, hematologic parameters (platelets, LDH, hemoglobin), and quality of life measures remained constant during both the 26-week initial evaluation period and through all available follow-up. All patients maintained the same CKD stage as recorded at baseline throughout the study, and no patient required dialysis at baseline or at any point throughout this study.

There were no unexpected safety findings or new safety signals identified through the current data-cut (median follow-up of 50.2 weeks (49.4–58.7 weeks)). The most frequent treatment-emergent AEs were upper respiratory tract infection and oropharyngeal pain. No serious AEs were found to be treatment related or necessitated treatment discontinuation, no meningococcal infections or deaths occurred during the trial, and no evidence of immunogenicity was identified. In previous trials conducted with eculizumab in pediatric patients with aHUS [8], 91% of patients experienced any AE, 41% had treatment-related AEs, and 59% had serious AEs. In the current study with ravulizumab, 20% had treatment-related AEs and 10% had serious AEs. No head-to-head trials of ravulizumab and eculizumab have been conducted, and differences in trial designs between the eculizumab and ravulizumab clinical studies may limit the comparability of their results.

A reduction in dosing frequency is an important element in improving the quality of life in patients with aHUS [17]; indeed, frequency of infusions has been shown to be one of the most important factors for patients switching to ravulizumab [23]. Based on the available data from this study, patients who switch to ravulizumab treatment can expect a similar efficacy and safety profile to eculizumab, giving sustained protection against TMA caused by aHUS with a longer interval between infusions. Recent data show that less frequent dosing with ravulizumab compared with eculizumab reduces the time spent in treatment by a substantial margin. As a result, costs associated with lost productivity are 60% lower in the clinic and 73% lower at home with ravulizumab treatment, compared with eculizumab treatment [24]. Furthermore, some patients may no longer require vascular access via a port, avoiding its associated comorbidities. This suggests that health system and societal costs with ravulizumab therapy are likely to be reduced, and quality of life is likely to improve for both patients and caregivers as a result of increased leisure time availability [24].

Furthermore, a study comparing the preference for eculizumab and ravulizumab among patients with paroxysmal nocturnal hemoglobinuria reported that ravulizumab was widely preferred, with frequency of infusions found to be the most important factor to patients [23]. A meta-analysis reported that patients with chronic diseases were more likely to adhere to their medication if the regimen was less frequent [25], while a systematic review also found that a reduced dosing frequency led to improvements in adherence, patient quality of life, patient satisfaction, and reduced costs [26].

However, it is important to highlight that, although ravulizumab and eculizumab target the same epitope of complement C5 protein, ravulizumab is a separate entity with distinct biochemical characteristics. In this study, ravulizumab treatment was initiated at the end of the last eculizumab dosing interval (14 days after the last eculizumab dose) with a weight-based loading dose, followed by weight-based maintenance dosing beginning 2 weeks post loading dose. Administration of a loading dose achieves immediate steady-state therapeutic concentrations of ravulizumab, ensuring complete and sustained terminal complement inhibition immediately upon switching to ravulizumab.

The main limitation of this study was a limited sample size. Due to the rarity of aHUS, study enrolment was restricted, and the sample size of this study was relatively small, meaning that the overall dataset is more sensitive to outliers and skewed distribution. Further, pediatric GFR values change substantially within the first 5–10 years of life, for example, almost doubling between birth and 2.5 years. One patient in our analysis was < 2 years of age so the data should be cautiously interpreted.

## Conclusions

In pediatric patients with aHUS, switching treatment from eculizumab to ravulizumab administered every 4 to 8 weeks resulted in sustained maintenance of stable kidney and hematologic parameters through 1 year, with no unexpected safety concerns. The results obtained in this study indicate that it is possible for pediatric patients with aHUS to receive treatment less frequently with a dosing interval up to 8 weeks without compromising efficacy and safety.

## Electronic supplementary material

Supplementary Figure 1 (DOCX 310 kb)

Supplementary Figure 2 (DOCX 96 kb)

Supplementary Figure 3 (DOCX 48 kb)

Supplementary Figure 4 (DOCX 75 kb)

Supplementary Table 1 (DOCX 26 kb)

Supplementary Table 2 (DOCX 24 kb)

ESM 1 (PPTX 807 kb)

## Data Availability

Alexion will consider requests for disclosure of clinical study participant-level data provided that participant privacy is assured through methods like data de-identification, pseudonymization, or anonymization (as required by applicable law) and if such disclosure was included in the relevant study informed consent form or similar documentation. Qualified academic investigators may request participant-level clinical data and supporting documents (statistical analysis plan and protocol) pertaining to Alexion-sponsored studies. Further details regarding data availability and instructions for requesting information are available in the Alexion Clinical Trials Disclosure and Transparency Policy at http://alexion.com/research-development. Link to Data Request Form (https://alexion.com/contact-alexion/medical-information).

## References

[1] Fakhouri F, Zuber J, Fremeaux-Bacchi V, Loirat C (2017). Haemolytic uraemic syndrome. Lancet.

[2] Campistol JM, Arias M, Ariceta G, Blasco M, Espinosa L, Espinosa M, Grinyo JM, Macia M, Mendizabal S, Praga M, Roman E, Torra R, Valdes F, Vilalta R, Rodriguez de Cordoba S (2015). An update for atypical haemolytic uraemic syndrome: diagnosis and treatment. A consensus document. Nefrologia.

[3] Brodsky RA (2015). Complement in hemolytic anemia. Blood.

[4] Fremeaux-Bacchi V, Fakhouri F, Garnier A, Bienaime F, Dragon-Durey MA, Ngo S, Moulin B, Servais A, Provot F, Rostaing L, Burtey S, Niaudet P, Deschenes G, Lebranchu Y, Zuber J, Loirat C (2013). Genetics and outcome of atypical hemolytic uremic syndrome: a nationwide French series comparing children and adults. Clin J Am Soc Nephrol.

[5] Noris M, Caprioli J, Bresin E, Mossali C, Pianetti G, Gamba S, Daina E, Fenili C, Castelletti F, Sorosina A, Piras R, Donadelli R, Maranta R, van der Meer I, Conway EM, Zipfel PF, Goodship TH, Remuzzi G (2010). Relative role of genetic complement abnormalities in sporadic and familial aHUS and their impact on clinical phenotype. Clin J Am Soc Nephrol.

[6] EMA (2011) EU/3/09/653. Available at: https://www.ema.europa.eu/en/medicines/human/orphan-designations/eu309653. Accessed Jan 2019.

[7] FDA (2011) Eculizumab (Soliris). Available at: http://wayback.archive-it.org/7993/20170113081126/http://www.fda.gov/AboutFDA/CentersOffices/OfficeofMedicalProductsandTobacco/CDER/ucm273089.htm. Accessed March 2020.

[8] Greenbaum LA, Fila M, Ardissino G, Al-Akash SI, Evans J, Henning P, Lieberman KV, Maringhini S, Pape L, Rees L, van de Kar NC, Vande Walle J, Ogawa M, Bedrosian CL, Licht C (2016). Eculizumab is a safe and effective treatment in pediatric patients with atypical hemolytic uremic syndrome. Kidney Int.

[9] Legendre CM, Licht C, Muus P, Greenbaum LA, Babu S, Bedrosian C, Bingham C, Cohen DJ, Delmas Y, Douglas K, Eitner F, Feldkamp T, Fouque D, Furman RR, Gaber O, Herthelius M, Hourmant M, Karpman D, Lebranchu Y, Mariat C, Menne J, Moulin B, Nurnberger J, Ogawa M, Remuzzi G, Richard T, Sberro-Soussan R, Severino B, Sheerin NS, Trivelli A, Zimmerhackl LB, Goodship T, Loirat C (2013). Terminal complement inhibitor eculizumab in atypical hemolytic-uremic syndrome. N Engl J Med.

[10] Licht C, Greenbaum LA, Muus P, Babu S, Bedrosian CL, Cohen DJ, Delmas Y, Douglas K, Furman RR, Gaber OA, Goodship T, Herthelius M, Hourmant M, Legendre CM, Remuzzi G, Sheerin N, Trivelli A, Loirat C (2015). Efficacy and safety of eculizumab in atypical hemolytic uremic syndrome from 2-year extensions of phase 2 studies. Kidney Int.

[11] Fakhouri F, Hourmant M, Campistol JM, Cataland SR, Espinosa M, Gaber AO, Menne J, Minetti EE, Provot F, Rondeau E, Ruggenenti P, Weekers LE, Ogawa M, Bedrosian CL, Legendre CM (2016). Terminal complement inhibitor eculizumab in adult patients with atypical hemolytic uremic syndrome: a single-arm, open-label trial. Am J Kidney Dis.

[12] Menne J, Delmas Y, Fakhouri F, Licht C, Lommele A, Minetti EE, Provot F, Rondeau E, Sheerin NS, Wang J, Weekers LE, Greenbaum LA (2019). Outcomes in patients with atypical hemolytic uremic syndrome treated with eculizumab in a long-term observational study. BMC Nephrol.

[13] Ito S, Hidaka Y, Inoue N, Kaname S, Kato H, Matsumoto M, Miyakawa Y, Mizuno M, Okada H, Shimono A, Matsuda T, Maruyama S, Fujimura Y, Nangaku M, Kagami S (2019). Safety and effectiveness of eculizumab for pediatric patients with atypical hemolytic-uremic syndrome in Japan: interim analysis of post-marketing surveillance. Clin Exp Nephrol.

[14] Sheridan D, Yu ZX, Zhang Y, Patel R, Sun F, Lasaro MA, Bouchard K, Andrien B, Marozsan A, Wang Y, Tamburini P (2018). Design and preclinical characterization of ALXN1210: a novel anti-C5 antibody with extended duration of action. PLoS One.

[15] FDA (2018). Ravulizumab prescribing information.

[16] EMA (2019). Ravulizumab summary of product characteristics.

[17] Rondeau E, Scully M, Ariceta G, Barbour T, Cataland S, Heyne N, Miyakawa Y, Ortiz S, Swenson E, Vallee M, Yoon SS, Kavanagh D, Haller H, 311 Study Group (2020). The long-acting C5 inhibitor, ravulizumab, is effective and safe in adult patients with atypical hemolytic uremic syndrome naive to complement inhibitor treatment. Kidney Int.

[18] Schwartz GJ, Muñoz A, Schneider MF, Mak RH, Kaskel F, Warady BA, Furth SL (2009). New equations to estimate GFR in children with CKD. J Am Soc Nephrol.

[19] Dragon-Durey MA, Blanc C, Roumenina LT, Poulain N, Ngo S, Bordereau P, Frémeaux-Bacchi V (2014). Anti-factor H autoantibodies assay. Methods Mol Biol.

[20] Kidney Disease Improving Global Outcomes (2013). KDIGO 2012 clinical practice guideline for the evaluation and management of chronic kidney disease. Kidney Int Suppl.

[21] Caprioli J, Bettinaglio P, Zipfel PF, Amadei B, Daina E, Gamba S, Skerka C, Marziliano N, Remuzzi G, Noris M (2001). The molecular basis of familial hemolytic uremic syndrome: mutation analysis of factor H gene reveals a hot spot in short consensus repeat 20. J Am Soc Nephrol.

[22] Caprioli J, Noris M, Brioschi S, Pianetti G, Castelletti F, Bettinaglio P, Mele C, Bresin E, Cassis L, Gamba S, Porrati F, Bucchioni S, Monteferrante G, Fang CJ, Liszewski MK, Kavanagh D, Atkinson JP, Remuzzi G (2006). Genetics of HUS: the impact of MCP, CFH, and IF mutations on clinical presentation, response to treatment, and outcome. Blood.

[23] Peipert JD, Kulasekararaj AG, Gaya A, Langemeijer SMC, Yount S, Fernandez FAG, Gutierrez EO, Martens C, Sparling A, Webster KA, Cella D, Tomazos I, Ogawa M, Piatek CI, Wells R, Hill A, Kaiser K (2019). PF734 patient preferences for the treatment of paroxysmal nocturnal hemoglobinuria: results of a patient survey of ravulizumab (ALXN1210) and eculizumab. HemaSphere.

[24] Levy A, Chen PGF, Tomazos I (2019). PRO5 comparing productivity losses from treating atypical hemolytic uremic syndrome patients in the united states with eculizumab or ravulizumab in an infusion clinic or at home. Value Health.

[25] Coleman CI, Limone B, Sobieraj DM, Lee S, Roberts MS, Kaur R, Alam T (2012). Dosing frequency and medication adherence in chronic disease. J Manag Care Pharm.

[26] Richter A, Anton SF, Koch P, Dennett SL (2003). The impact of reducing dose frequency on health outcomes. Clin Ther.

