# Supplementary material for: The long-acting C5 inhibitor, ravulizumab, is efficacious and safe in pediatric patients with atypical hemolytic uremic syndrome previously treated with eculizumab
Source: Pediatr Nephrol. 2020 Oct 13;36(4):889–98. doi: 10.1007/s00467-020-04774-2 (PMC7910247; doi:10.1007/s00467-020-04774-2)
Supplement: Supplementary file 1 — (DOCX 310 kb) [file 467_2020_4774_MOESM1_ESM.docx]

**The long-acting C5 inhibitor, ravulizumab, is efficacious and safe in pediatric patients with atypical hemolytic uremic syndrome previously treated with eculizumab**

**Pediatric Nephrology**

Dr. Kazuki Tanaka,^1^ Dr. Brigitte Adams,^2^ Dr. Alvaro Madrid Aris,^3^ Dr. Naoya Fujita,^1^ Dr. Masayo Ogawa,^4^ Dr. Stephan Ortiz,^4^ Mr. Marc Vallee,^4^ Dr. Larry A. Greenbaum^5^

Corresponding author:

Dr Kazuki Tanaka

Head Physician, Department of Nephrology, Aichi Children's Health and Medical Center

Postcode: 474-8710 7-426, Morioka-cho, Obu City, Aichi prefecture, Japan

Tel: +81-562-43-0500


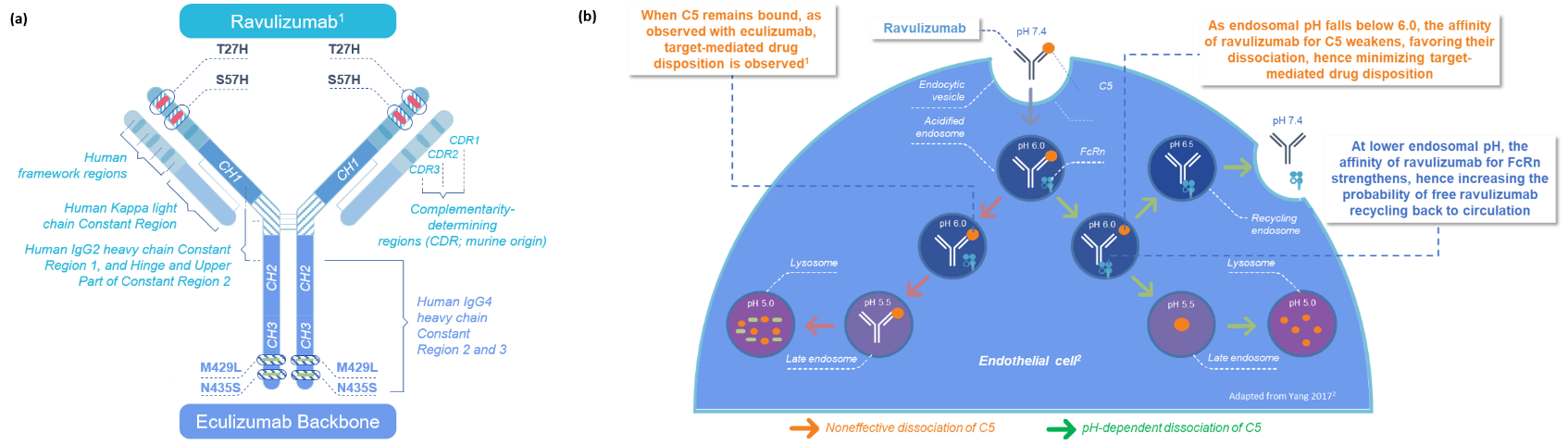
E-mail: kazuki.tanaka0505@gmail.com

**Supplementary Fig. 1** Ravulizumab molecule schematic, including amino acid substitutions base on the eculizumab molecular scaffold (a) and a graphical representation of the mechanism of ravulizumab half-life prolongation (b). The replacement of Tyr-27 and Ser-57 with His residues in the variable heavy chain CDR-1 and -2 regions accelerate pH-dependent dissociate from complement C5 in acidified endosomes, reducing the affinity for C5 at pH 6.0 by a factor of 36 with only a minimal impact at pH 7.4. The replacement of Met-429 with Leu and Asn-435 with Ser in the heavy chain CH3 domain enhances pH-dependent binding to FcRn by a factor of 10.

Asn, Asparagine; CDR, Complementarity-determining region; CH, Constant domain; FcRn, Neonatal FC receptor; Leu, Leucine; His, Histidine; Met, Methionine; Ser, Serine; Tyr, Tyrosine
