# Supplementary material for: The long-acting C5 inhibitor, ravulizumab, is efficacious and safe in pediatric patients with atypical hemolytic uremic syndrome previously treated with eculizumab
Source: Pediatr Nephrol. 2020 Oct 13;36(4):889–98. doi: 10.1007/s00467-020-04774-2 (PMC7910247; doi:10.1007/s00467-020-04774-2)
Supplement: Supplementary file 2 — (DOCX 96 kb) [file 467_2020_4774_MOESM2_ESM.docx]

**The long-acting C5 inhibitor, ravulizumab, is efficacious and safe in pediatric patients with atypical hemolytic uremic syndrome previously treated with eculizumab**

**Pediatric Nephrology**

Dr. Kazuki Tanaka,^1^ Dr. Brigitte Adams,^2^ Dr. Alvaro Madrid Aris,^3^ Dr. Naoya Fujita,^1^ Dr. Masayo Ogawa,^4^ Dr. Stephan Ortiz,^4^ Mr. Marc Vallee,^4^ Dr. Larry A. Greenbaum^5^

Corresponding author:

Dr Kazuki Tanaka

Head Physician, Department of Nephrology, Aichi Children's Health and Medical Center

Postcode: 474-8710 7-426, Morioka-cho, Obu City, Aichi prefecture, Japan

Tel: +81-562-43-0500

E-mail: kazuki.tanaka0505@gmail.com


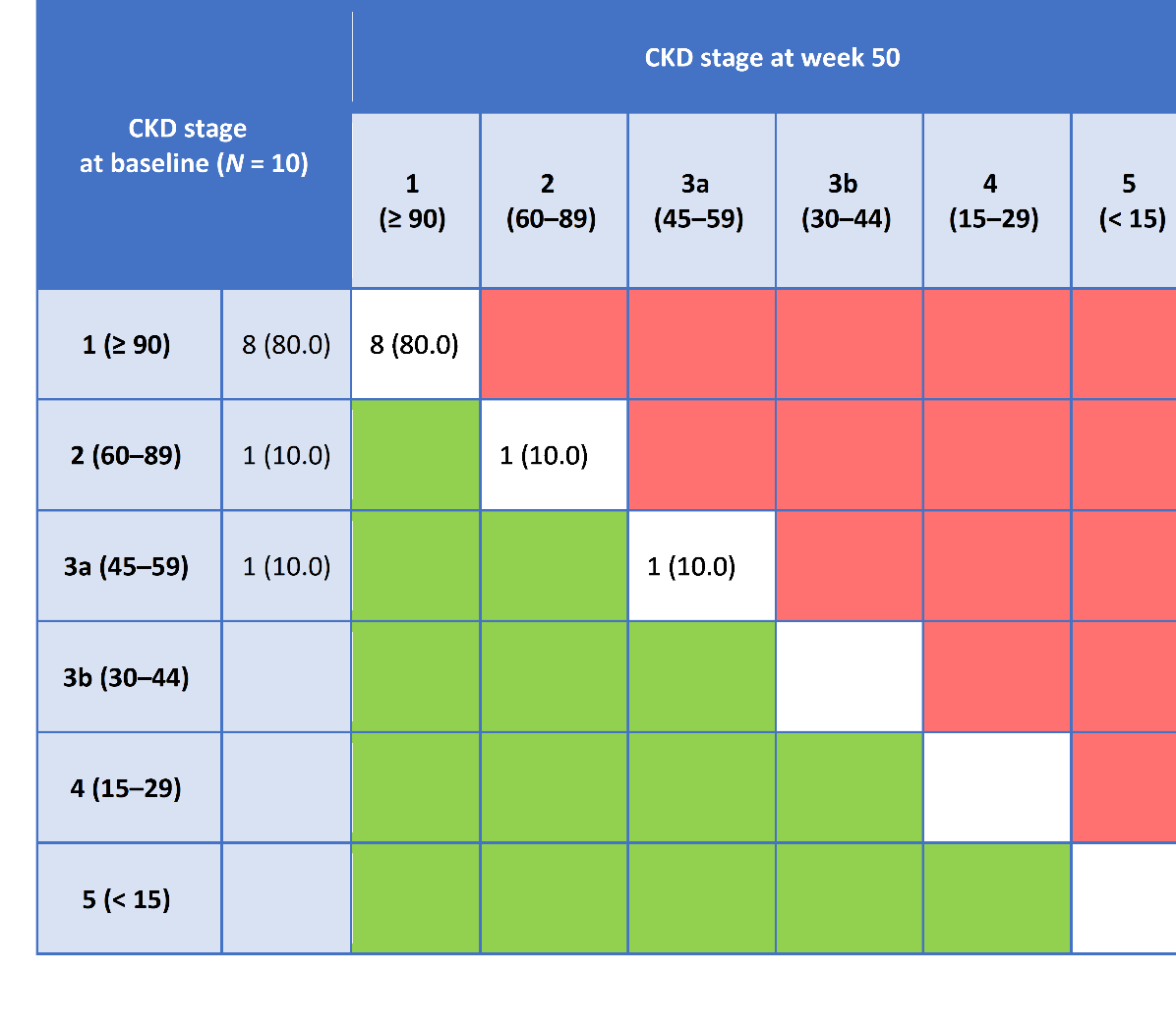


**Supplementary Fig. 2** Shift in CKD stage category from baseline to 52 weeks (full analysis set). Data shown as n (%). CKD stages are shown with corresponding eGFR ranges in ml/min per 1.73 m^2^. Green cells represent improvement from baseline to 26 weeks; red cells represent worsening, and white cells represent no change. *CKD* chronic kidney disease, *eGFR* estimated glomerular filtration rate
