# Supplementary material for: The long-acting C5 inhibitor, ravulizumab, is efficacious and safe in pediatric patients with atypical hemolytic uremic syndrome previously treated with eculizumab
Source: Pediatr Nephrol. 2020 Oct 13;36(4):889–98. doi: 10.1007/s00467-020-04774-2 (PMC7910247; doi:10.1007/s00467-020-04774-2)
Supplement: Supplementary file 5 — (DOCX 26 kb) [file 467_2020_4774_MOESM5_ESM.docx]

**The long-acting C5 inhibitor, ravulizumab, is efficacious and safe in pediatric patients with atypical hemolytic uremic syndrome previously treated with eculizumab**

**Pediatric Nephrology**

Dr. Kazuki Tanaka,^1^ Dr. Brigitte Adams,^2^ Dr. Alvaro Madrid Aris,^3^ Dr. Naoya Fujita,^1^ Dr. Masayo Ogawa,^4^ Dr. Stephan Ortiz,^4^ Mr. Marc Vallee,^4^ Dr. Larry A. Greenbaum^5^

Corresponding author:

Dr Kazuki Tanaka

Head Physician, Department of Nephrology, Aichi Children's Health and Medical Center

Postcode: 474-8710 7-426, Morioka-cho, Obu City, Aichi prefecture, Japan

Tel: +81-562-43-0500

E-mail: kazuki.tanaka0505@gmail.com

**Supplementary Table 1** Full phase III pediatric ravulizumab clinical study inclusion and exclusion criteria

| **Inclusion Criteria** | **Exclusion Criteria** |
| --- | --- |
| Patients from birth up to < 18 years of age and weighing ≥ 5 kg at the time of consent | Known familial or acquired ‘a disintegrin and metalloproteinase with a thrombospondin type 1 motif, member 13’ (ADAMTS13) deficiency (activity < 5%) |
| For Cohort 1 patients, evidence of TMA, including thrombocytopenia, evidence of hemolysis, and kidney injury | Known Shiga toxin-related hemolytic uremic syndrome (ST-HUS) as demonstrated by a positive test for Shiga toxin or culture of Shiga toxin producing bacteria |
| For Cohort 2 patients, documented diagnosis of aHUS | Positive direct Coombs test |
| For Cohort 2 patients, clinical evidence of response to eculizumab indicated by stable TMA parameters (via central laboratory results) at Screening | Known HIV infection |
| Known history of aHUS prior to current kidney transplant **OR** No known history of aHUS, and persistent evidence of TMA at least 4 days after modifying the immunosuppressive regimen (eg, suspending or reducing the dose) of CNI (eg, cyclosporine, tacrolimus) or mTORi (eg, sirolimus, everolimus) | Unresolved meningococcal disease |
| Among patients with onset of TMA postpartum, persistent evidence of TMA for > 3 days after the day of childbirth | Patients with a confirmed diagnosis of ongoing sepsis defined as positive blood cultures within 7 days prior to the start of Screening and untreated with antibiotics |
| To reduce the risk of meningococcal infection (Neisseria meningitidis), all patients must be vaccinated against meningococcal infections within 3 years prior to, or at the time of, initiating study drug. Patients who receive a meningococcal vaccine less than 2 weeks before initiating ALXN1210 treatment must receive treatment with appropriate prophylactic antibiotics until 2 weeks after vaccination. Patients who have not been vaccinated prior to initiating ALXN1210 treatment should receive prophylactic antibiotics prior to and for at least 2 weeks after meningococcal vaccination. Patients who cannot be vaccinated must receive antibiotic prophylaxis for the entire treatment period and for 8 months following last dose | Presence or suspicion of active and untreated systemic bacterial infection that, in the opinion of the Investigator, confounds an accurate diagnosis of aHUS or impedes the ability to manage the aHUS disease |
| Patients must have been vaccinated against Hib and Streptococcus pneumoniae according to national and local vaccination schedule guidelines | Females who plan to become pregnant during the study or are currently pregnant or breastfeeding |
| Female patients of childbearing potential and male patients with female partners of childbearing potential must follow protocol-specified guidance for avoiding pregnancy while on treatment and for 8 months after last dose of study drug | Heart, lung, small bowel, pancreas, or liver transplant |
| Patient's legal guardian must be willing and able to give written informed consent and the patient must be willing to give written informed assent (if applicable as determined by the central or local IRB/IEC) and comply with the study visit schedule | Among patients with a kidney transplant, acute kidney dysfunction within 4 weeks of transplant consistent with the diagnosis of acute AMR according to Banff 2013 criteria |
|  | Among patients without a kidney transplant, history of kidney disease other than aHUS |
|  | Identified drug exposure-related HUS |
|  | For Cohort 1 patients, receiving PE/PI, for 28 days or longer, prior to the start of Screening for the current TMA |
|  | History of malignancy within 5 years of Screening with the exception of a non-melanoma skin cancer or carcinoma in situ of the cervix that has been treated with no evidence of recurrence |
|  | BMT/HSCT within the last 6 months prior to the start of Screening |
|  | HUS related known genetic defects of cobalamin C metabolism |
|  | Known systemic sclerosis (scleroderma), SLE, or antiphospholipid antibody positivity or syndrome |
|  | Chronic dialysis (defined as dialysis on a regular basis as renal replacement therapy for ESKD) |
|  | Patients receiving chronic mAb immunoglobulin (IVIg) within 8 weeks prior to the start of Screening, unless for unrelated medical condition (eg, hypogammaglobinemia); or chronic rituximab therapy within 12 weeks prior to the start of Screening |
|  | Patients receiving other immunosuppressive therapies such as steroids, mTORi (eg, sirolimus, everolimus), CNI (eg, cyclosporine or tacrolimus) are excluded unless:   1. part of an established post-transplant antirejection regimen, or 2. patient has confirmed anti-complement factor antibodies requiring immunosuppressive therapy, or 3. steroids are being used for a condition other than aHUS (eg, asthma). |
|  | Participation in another interventional treatment study or use of any experimental therapy within 30 days before initiation of study drug on Day 1 in this study or within 5 half-lives of that investigational product, whichever is greater |
|  | For Cohort 1 patients, prior use of any complement inhibitors |
|  | For Cohort 2 patients, prior use of complement inhibitors other than eculizumab |
|  | For Cohort 2 patients, any known abnormal TMA parameters within 90 days prior to Screening (ie, LDH ≥ 1.5 X ULN, or platelet count < 150,000/μL, or eGFR ≤ 30 mL/min/1.73m^2^ using the Schwartz formula) |
|  | Hypersensitivity to any ingredient contained in the study drug, including hypersensitivity to murine proteins |
|  | Any medical or psychological condition that, in the opinion of the Investigator or Sponsor, could increase the risk to the patient by participating in the study or confound the outcome of the study |
|  | Known or suspected history of drug or alcohol abuse or dependence within 1 year prior to the start of Screening |
|  | Use of tranexamic acid within 7 days prior to Screening is prohibited |

Cohort 1: eculizumab naïve patient population; Cohort 2: previously treated with eculizumab population

*aHUS* atypical hemolytic uremic syndrome, *AMR* antibody-mediated rejection, *BMT* bone marrow transplant, *CNI* calcineurin inhibitor, *eGFR* estimated flomerular filtration rate, *ESKD* end-stage kidney disease, *Hib* Haemophilus influenzae type b, *HIV* human immunodeficiency virus, *HSCT* hematopoietic stem cell transplant, *IEC* Institutional (or Independent) Ethics Committee, *IRB* Institutional Review Board, *LDH* lactate dehydrogenase, *mAb* humanized monoclonal antibody, *mTORi* mammalian target of rapamycin inhibitor, *PE* plasma exchange, *PI* plasma infusion, *SLE* systemic lupus erythematosus, *ST-HUS* Shiga toxin-related hemolytic uremic syndrome, *TMA* thrombotic microangiopathy, *ULN* upper limit of normal
