# Supplementary material for: The long-acting C5 inhibitor, ravulizumab, is efficacious and safe in pediatric patients with atypical hemolytic uremic syndrome previously treated with eculizumab
Source: Pediatr Nephrol. 2020 Oct 13;36(4):889–98. doi: 10.1007/s00467-020-04774-2 (PMC7910247; doi:10.1007/s00467-020-04774-2)
Supplement: Supplementary file 6 — (DOCX 24 kb) [file 467_2020_4774_MOESM6_ESM.docx]

**The long-acting C5 inhibitor, ravulizumab, is efficacious and safe in pediatric patients with atypical hemolytic uremic syndrome previously treated with eculizumab**

**Pediatric Nephrology**

Dr. Kazuki Tanaka,^1^ Dr. Brigitte Adams,^2^ Dr. Alvaro Madrid Aris,^3^ Dr. Naoya Fujita,^1^ Dr. Masayo Ogawa,^4^ Dr. Stephan Ortiz,^4^ Mr. Marc Vallee,^4^ Dr. Larry A. Greenbaum^5^

Corresponding author:

Dr Kazuki Tanaka

Head Physician, Department of Nephrology, Aichi Children's Health and Medical Center

Postcode: 474-8710 7-426, Morioka-cho, Obu City, Aichi prefecture, Japan

Tel: +81-562-43-0500

E-mail: kazuki.tanaka0505@gmail.com

**Supplementary Table 2** Patient genetics data. All study investigators were requested to provide retrospective genetic screening data, where available. The genetic testing methodologies at facilities used by individual clinicians may vary and have not been independently verified. Therefore, the study sponsor assumes no responsibility for the accuracy and consistency of these data. In all, 8/10 study investigators responded with genetic information and 3 more patients were identified as carriers of pathogenic variants in genes implicated in the pathogenesis of aHUS (complement factor B [CFB], MCP/CD46 and complement C3), bringing the total number of patients carrying pathogenic variants to 5 of the 9 patients tested (55%).

| **Patient ID** | **Clinician genetics findings summary *** | **NCT03131219 Trial Genetics**** | **Classification^‖^** | **NCT03131219 Trial CFH antibody test positivity** |
| --- | --- | --- | --- | --- |
| 1 | Missense mutation K350N on exon 8 of CFB gene | Not tested | Pathogenic variant | Negative |
| 2 | No pathogenic variant | Not tested | No pathogenic variant | Negative |
| 3 | Missense mutation Y189D on SCR3 domain of MCP gene | Not tested | Pathogenic variant | Negative |
| 4 | No pathogenic variant | Not tested | No pathogenic variant | Negative |
| 5 | Not tested | Nonsense variant c.175C>T (p.Arg59Ter) in exon 2 (SCR1 domain) of member cofactor protein (MCP, CD46) gene | pathogenic | Negative |
| 6 | No pathogenic variant | No pathogenic variant detected | No pathogenic variant | Negative |
| 7 | Missense variant c.3644G>A,p.Arg1215Gin on CFH gene | Missense variant c.3644G>A (p.Arg1215Gln) on exon 22 (SCR20 domain) of Complement Factor H (CFH) gene | Pathogenic | Negative |
| 8 | Heterozygous missense variant c.3124C>G (p.Arg1042Gly) on C3 gene | Not tested | Pathogenic | Negative |
| 9 | No response form clinician | Not tested | N/A | Negative |
| 10 | No response form clinician | No pathogenic variant detected | No pathogenic variant | Positive |

*Data collected outside of this clinical trial (NCT03131219) and provided by investigators as response to request by the trial sponsor, ** Genetic testing carried out in trial patients who consented Classification based on either clinician genetics findings and/or this trial (NCT03131219).
