# Supplementary material for: The long-acting C5 inhibitor, ravulizumab, is efficacious and safe in pediatric patients with atypical hemolytic uremic syndrome previously treated with eculizumab
Source: Pediatr Nephrol. 2020 Oct 13;36(4):889–98. doi: 10.1007/s00467-020-04774-2 (PMC7910247; doi:10.1007/s00467-020-04774-2)
Supplement: Supplementary file 7 — (PPTX 807 kb) [file 467_2020_4774_MOESM7_ESM.pptx]

## Slide 1
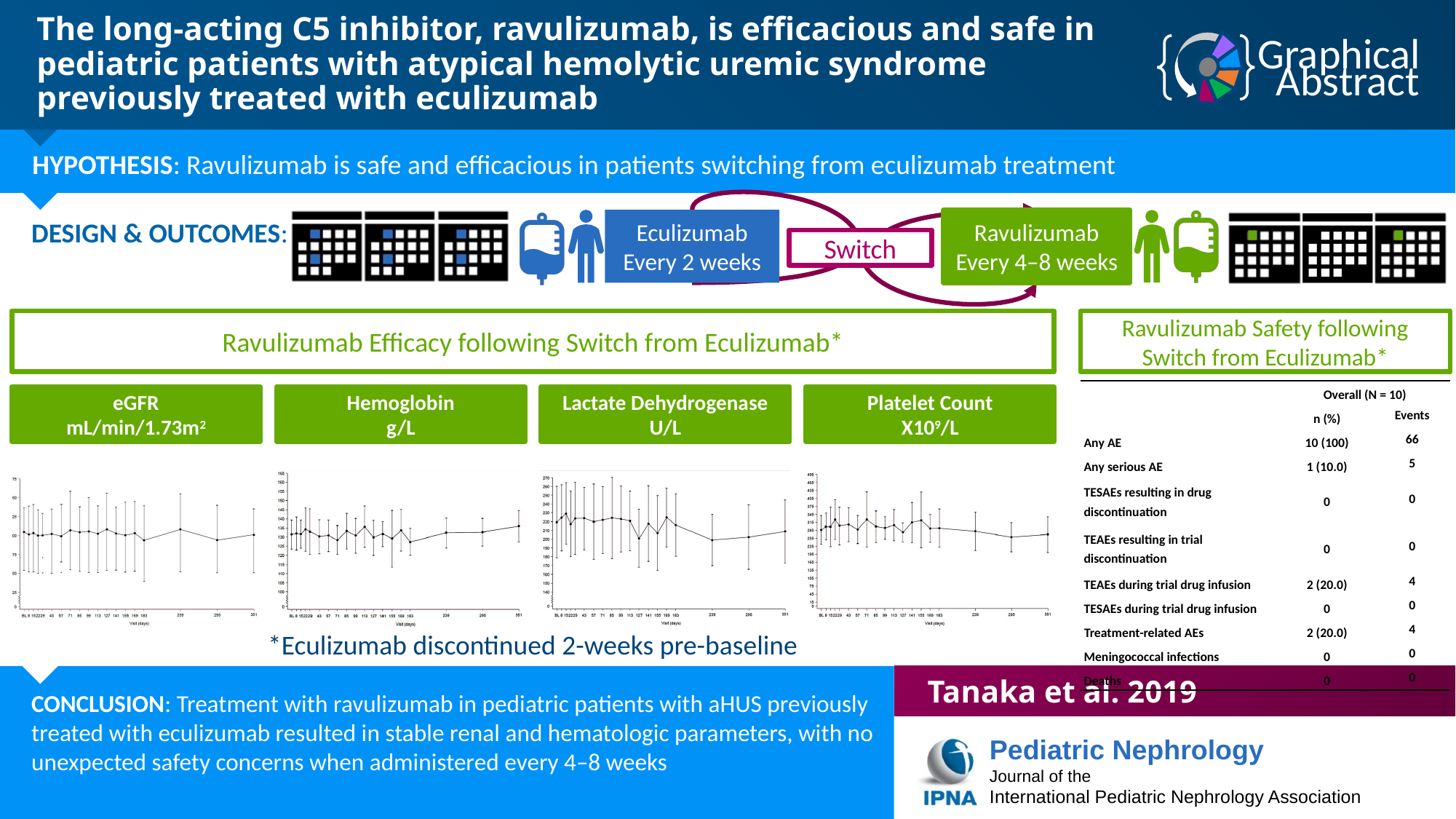

The long-acting C5 inhibitor, ravulizumab, is efficacious and safe in pediatric patients with atypical hemolytic uremic syndrome previously treated with eculizumab
HYPOTHESIS: Ravulizumab is safe and efficacious in patients switching from eculizumab treatment
Eculizumab
Every 2 weeks
Ravulizumab
Every 4–8 weeks
Switch
DESIGN & OUTCOMES:
Ravulizumab Efficacy following Switch from Eculizumab*
Ravulizumab Safety following Switch from Eculizumab*
| | Overall (N = 10) | |
| --- | --- | --- |
| | n (%) | Events |
| Any AE | 10 (100) | 66 |
| Any serious AE | 1 (10.0) | 5 |
| TESAEs resulting in drug discontinuation | 0 | 0 |
| TEAEs resulting in trial discontinuation | 0 | 0 |
| TEAEs during trial drug infusion | 2 (20.0) | 4 |
| TESAEs during trial drug infusion | 0 | 0 |
| Treatment-related AEs | 2 (20.0) | 4 |
| Meningococcal infections | 0 | 0 |
| Deaths | 0 | 0 |
eGFR
mL/min/1.73m2
Hemoglobin
g/L
Lactate Dehydrogenase
U/L
Platelet Count
X109/L
*Eculizumab discontinued 2-weeks pre-baseline
Tanaka et al. 2019
CONCLUSION: Treatment with ravulizumab in pediatric patients with aHUS previously treated with eculizumab resulted in stable renal and hematologic parameters, with no unexpected safety concerns when administered every 4–8 weeks
